# Progress in Medicine

**Published:** 1899-05-13

**Authors:** 


### Progress in Medicine.

### DISEASES OF THE NERVOUS SYSTEM.

(*Continued from page* 97.)

**Disturbances of Sensation in Disease of the Nervous System.**—Williamson[10] records a series of cases of disease of the nervous system in which disturbances of sensation occurred. As is well known, complete hemianæsthesia is due either to hysteria or to a lesion of the interna capsule; but it is not generally recognised that hemianæsthesia due to an organic lesion may in some cases terminate abruptly in one limb, *e.g.*, at the calf or ankle, and on the part below sensation may be quite normal. In other undoubtedly organic cases the anæsthesia may affect the whole of one side, with exception of the face and neck; again, the limbs may be more anæsthetic than the trunk or face. And in the progress of recovery the return of sensation may be in the limbs from above downwards. Lesions of the crus may also produce hemianæsthesia, when in the pons there may be crossed anæsthesia, *i.e.*, of face on one side and of the opposite side of the body. When hemiplegia and hemianæsthesia from cerebral disease are well marked, there is loss of sensation to both tactile and painful sensation along with thermoanæsthesia and loss of muscular sense; when it is slight there may be loss of sensation to tactile but not to painful impressions.

It is well-known that a lesion limited to the motor area of the cortex never gives rise to a marked and complete hemianæsthesia. Cases are on record where such lesions produced diminution of sensation or anæsthesia chiefly localised to one limb and nearly at its distal extremity. And on the other hand there may be extensive destruction of the motor cortex without any anæsthesia.

In cases of marked anæsthesia of the hand, the blindfolded patient is, of course, unable to recognise the nature of objects placed in the hand. Cases also occasionally occur in which there is but little or no tactile anæsthesia, and yet the patient is quite unable to tell the nature of the objects placed in the hand, unable, as one patient graphically said, "to picture it." This "touch paralysis," as it has been called, is analogous with word blindness and word deafness, where words are seen or heard and yet their meaning is not appreciated. The lesion producing this "touch paralysis" is possibly in the parietal region behind the motor arm centre, but the pathological evidence is as yet not conclusive.

In cerebral disease, when there is loss of sensation in a portion of a limb only, the boundary line of the anæsthesia is more or less at right angles to the long axis of the limb. On the other hand, in spinal diseases, when there is anæsthesia in a portion of a limb, only the boundary line of the anæsthesia runs more or less parallel with the long axis of the limb.

In some cases of spinal disease there is loss of sensation to tactile impressions, while sensation for painful impressions and the sense of temperature are unaffected. Or it may be exactly the reverse: now this latter occurs in many cases of syringomyelia, and is strong evidence in favour of that diagnosis if of slow development. This loss of sensation to pain and temperature without tactile anæsthesia may, however, occur in other spinal cord diseases, *e.g.*, syphilitic paraplegia, spinal hæmorrhage, spinal tumour, acute poliomyelitis.

Thermoanæsthesia may be the only form of sensory affection in certain spinal diseases, especially spinal syphilis; its presence excludes at once those spinal diseases which produce motor symptoms only, and is very strong evidence against hysteria, since in that disease the symptom very rarely occurs, In peripheral neuritis from causes other than lead there is usually some affection of sensation, though this as a rule is less marked than the motor disturbance. Muscular hyperæsthesia with some cutaneous anæsthesia is often met with. Or there may be tactile anæsthesia combined with hyperæsthesia for painful impressions, or the reverse.

**Retinal Changes in Cerebral Hæmorrhage, Embolism, and Thrombosis.**—It is well known that in a large number of cases of cerebral hæmorrhage, ophthalmoscopic examination reveals albuminuric retinitis in each eye. And in cerebral embolism from ulcerative endocarditis, hæmorrhage and white patches may sometimes be found in both eyes and occasionally embolism of the central artery of the retina. But unilateral changes in the retina are less well-known. Williamson[11] has recorded such cases. He finds that in cases of hemiplegia from cerebral hæmorrhage which terminate fatally, large hæmorrhages are not unfrequently found in the retina on the same side as the lesion, whilst no hæmorrhages are present in the opposite retina. In cerebral embolism the same retinal condition is occasionally met with; also in cerebral embolism the retinal vessels are occasionally slightly dilated on the side of the brain lesion. In thrombosis of the middle cerebral artery, when the thrombus extends down into the internal carotid, the vessels on the side of the brain lesion may be markedly dilated and tortuous, whilst the retinal vessels of the other side are normal.

Our knowledge of family spastic paraplegia has recently been summarised by Lorraine.[12] Nervous and mental diseases, syphilis, alcoholism, and relationship between father and mother predispose to the disease, which is found to affect the sexes equally. The typical symptoms are spastic gait, with increased reflexes and ankle clonus without sensory impairment or interference with the sphincters. Occasionally optic atrophy is observed. Rarely intelligence is defective. The course is usually one of gradually increasing interference with working power, and death is often the result of tuberculosis. The only necropsy has been reported by Strümpell, who found primary combined sclerosis of the pyramidal tracts, of the direct cerebellar tracts, and of the column of Goll. Myelitis, spinal syphitis, and multiple sclerosis are to be distinguished by the absence of sensory and sphincter impairment, and the presence of the family history. Hereditary cerebellar ataxy presents some features of similarity, but is sufficiently distinguished by the ataxy of the gait. In regard to treatment, warm baths, massage, and rest are recommended, and in certain cases tenotomy may occasionally be useful.

**Primary Focal Hæmatomyelia from Traumatism** was first described by Thornburn, and more recently Minor and Bailey[13] have recorded cases. By the term is meant localised hæmorrhage into the substance of the spinal cord as a result of injury. It is to be distinguished from what may be called secondary traumatic hæmatomyelia in which the hæmorrhages simply complicate crushes of the spinal cord from fracture-dislocation of the vertebræ. In the primary variety demonstrable lesions of the bones are absent, and the hæmorrhage accordingly constitutes the chief lesion, being uncomplicated by general mutilation of the cord. The affection is certainly more frequent than is generally recognised, yet out of 21 fatal cases of spinal cord traumatisms examined by Thornburn six were interpreted as instances of hæmatomyelia. And the percentage of primary cases as shown by autopsy is by no means expressive of their relative frequency, because this is the form of injury to the spinal cord in which recovery most often occurs. The most prominent post-mortem feature in the spinal cord at a post-mortem examination is the hæmorrhage. Laceration of the nerve fibres probably also takes place, but it is so difficult to detect that we have to rely on the amount of hæmorrhage as an indication of the amount of injury. The blood is poured out first and most profusely in the grey matter of the cord, which is often marked out in red as a result. This total occupation of the grey matter by blood rarely extends to more than one or two segments, though small pencils of bloo extend upwards and downwards in the grey matter. If the hæmorrhage is profuse it may extend into the white matter at the focus of greatest injury. If recovery ensues the blood is gradually absorbed and a cavity left, which may be more or less filled up with new connective tissue. The cause of the affection results almost exclusively from sudden forced flexions or extensions of the neck; *e.g.*, falls on the head, dropping of heavy weights on the head, diving into shallow water. The hæmorrhage probably results from rupture of one or more of blood vessels of the grey matter, brought about by stretching of the cord during the forced flexion or extension of the head.

As regards the symptoms, there may be œdema, bruising, stiffness, pain in the neck due to the wrench, but crepitus, false motion, and pathological irregularities in the vertebral spines are conspicuous by their absence. The spinal cord symptoms, if the hæmorrhage involves the whole cord, are those of a total transverse lesion; but if, as is usually the case, the hæmorrhage exceeds the grey matter but little or not at all, the clinical picture has a distinct individuality, of which the most salient features are an initial motor paralysis which soon becomes modified, and anæsthesia for temperature or pain, or both, without impairment of the sense of touch. The motor paralysis ensues instantly on receipt of the injury. The muscles involved at the level of the injury (as the hæmorrhage is nearly always in the lower cervical region) are those of the forearm and hand, and sometimes of the upper arm. These rarely perfectly recover, as the corresponding cornual cells are much damaged or destroyed, leading to wasting of the muscles supplied. The legs are, as a rule, also paralysed from direct or indirect (through pressure) damage to the pyramidal tracts; but this condition is often in large part recovered from, and the resulting paralysis, if any, is of a spastic type. The disturbance of sensation is, as mentioned above, a dissociated anæsthesia, of the same type as occurs in syringomyelia. The facts of both diseases suggest that the passage of stimuli giving rise to sensations of pain and temperature pass up in the grey, those of touch in the white, matter of the cord. The degree of thermo-anæsthesia and analgesia is not constant; as a rule analgesia is the less of the two, and in recovery also it is the first to diminish and disappear. The tendency of these affections of the pain and temperature sense is towards improvement, and sometimes recovery is perfect. Sharp lancinating pains down the arms are not prominent in pure central traumatic hæmatomyelia, neither are numbness, tingling, or other forms of paræsthesias frequent. Myosis is frequent, as the hæmorrhages are in the vicinity of the cilio-spinal segment. The general symptoms of any traumatism of the spinal cord may be present, such as retention of urine, incontinence of fæces, distension of the abdomen, fever. They are, however, generally of short duration, and one or more of them may be absent.

The diagnosis is, as a rule, to be made by exclusion of any physical signs of fracture-dislocation of the vertebræ and by the presence of the peculiar dissociated anæsthesia. The most difficult cases are those in which the hæmorrhage is either very great or very small. The prognosis, where the diagnosis is clear, is very good as regards life, and it often happens that the only abiding symptom is the paralysis and wasting of some of the muscles of one or both arms.

[10] Williamson, Med. Chron., Feb., 1899. [11] Williamson, Brit. Med. Jour., June 11, 1898. [12] Lorraine, Neurol. Central, 1898. [13] Bailey, Med. Rec., Nov. 19, 1898.

### FEVERS.

**Malaria.**—T. Ewing's[1] conclusions from a study of 800 cases in the late war are (*a*) that typhoid is to a large extent incompatible with active malarial fever, and during the course of the former the latter is usually suppressed, active sporulation rarely occurring during typhoid; (*b*) that old malaria may alter the course of typhoid through the anæmia; (*c*) that malarial paroxysms often occur during convalescence, so that some germs must persist through the typhoid fever, and it is possible that irregularities of temperature may be due to this suppressed growth. Lyon,[2] in discussing the typho-malarial fever as described by American writers since 1862, which is a much milder disease than typhoid, and often regarded as a hybrid form, thinks that true cases of combined infection do occur, but these are not the ones which are so constantly reported in the Southern States. Thus, no less than 57,400 instances were reported in the Civil War, and to-day the name is given to most fevers which present any difficulty in diagnosis In the Johns Hopkins Hospital typho-malarial is never met with, and only two cases of combined typhoid and malaria have been found, while Dock declares he has never met with a case of either. Similar opinions are reported from France and Italy, though a few cases of the combined affection have been recorded there which were marked by great severity and high mortality. Lyon analyses 30 instances of combined infection, and shows that they present a very different picture to that of the common "typho-malaria," which he thinks is either mild typhoid or pure malaria.

**Treatment.**—W. H. Thomson,[3] in treating a hundred cases of æstivo-autumnal type with irregular intermissions, found that quinine alone was unreliable, but that its effect was very greatly increased if opium, in the form of the camphorated tincture, and ginger, or other aromatics were added. As much as three grains of opium daily were thus given with great relief to the head symptoms and rapid general improvement. He quotes from the British Opium Commission the evidence of Sir William Roberts as to the value of anarcotin in malarial fevers, which showed a rapid arrest of the paroxysms when given in doses of one to three grains. A. Jacobi[4] finds that many intractable cases of malaria will get well under ergot, which forces the stagnating blood and its organisms out of the enlarged spleen. If paroxysms should in any case recur after the spleen has contracted it may be desirable to use quinine in addision. For recent and acute cases he would rely solely on quinine. Chiurici[5] argues that the spleen has no power of destroying bacterial toxins, for in animals whose spleens had been removed injections of toxins produced exactly the same symptoms as in normal ones.

As to the vexed question of the production of hæmoglobinuria by quinine, a summary by Woldert in the *Medical News* shows that Italian and Greek physicians generally believe in its causation in some cases by quinine, while the Americans refer it to the action of malaria, and give quinine vigorously as a remedy. Cross and Pakes[6] describe a case coming on after service on the Niger, and where quinine was only given after the hæmoglobinuria had commenced. As the quinine was increased the blood disappeared from the water. The blood contained plasmodia of the malignant type, while red corpuscles, as well as colouring matter, were found in the urine. The authors think that the disease is merely a complication of malaria just as hyperpyrexia is of rheumatism. Washbourn adds that in some cases of malaria the brain or intestines may be specially affected by the malarial poison, while here the stress falls on the kidneys or bone marrow, causing a rapid blood destruction. Crosse[7] points out elsewhere that blackwater fever comes on usually when the patient is broken down after several attacks of ordinary fever, that it is very rare before the end of the first six months of life in Africa, and uncommon after the end of three years there; and that usually the patient has lived near upturned soil. Finally, he has never known an attack in a person who has taken quinine regularly as a preventative of malaria, and he refers to many cases successfully treated with quinine by Macgregor, Moffat, and others. The assertion that the disease is produced by quinine is also shown to be doubtful, because the reported cases are very few and always in malarial subjects. In the malignant malarial fevers the corpuscles of the blood are ruptured before the hæmoglobin is completely destroyed, and in severe paroxysms as many as a third of the total number of corpuscles may thus die and set free their hæmoglobin, which is taken up by the liver in most cases. If then from any cause the action of the liver ceases hæmoglobinuria takes place at once.

The author goes on to argue that we have three or four factors to treat in each case—the malarial attack, the chronic malarial toxæmia, the sudden loss of hæmoglobin, and sometimes the secondary nephritis. He gives enough quinine to keep the blood free from parasites, and prefers to administer hypodermically 10 grains of the hydrochlorate or lactate three times a day for two days, to be reduced gradually to five grains twice daily during the entire attack if there is free excretion by the kidneys. For the toxæmia, loss of blood, and nephritis we have only ordinary remedies to choose from.

It was formerly said the disease did not occur in India, but A. Powell records 11 cases in Assam, and C. E. Seal adds five more. The latter[8] notices the absence in his patients of rigors pyrexia and sweating, which Powell observed in his. Moreover, though none of them had taken large doses of quinine before the attacks, the writer thinks that it did harm when he gave it during the outbreak.

**Snake Bite.**—Ch. J. Martin[9] agrees with Fraser's conclusion that it requires many times as much antivenene to counteract a given dose of poison when injected separately as it does when they are mixed prior to injection, but he finds from experiments of his own that if the antivenene is injected into the blood stream and the poison into subcutaneous tissue only a small quantity is needed. He therefore advises only intravenous injections in the treatment of snake-bite, believing that from the tissues the poison is absorbed more rapidly than the antidote. Semple and Ram,[10] working at Netley, find that when the serum is injected into the veins five minutes before the venom no more is required than when both are mixed beforehand, and assert that if intravenous injections are given within, say, half-an-hour of a snake-bite, the results will be the same as when prior injections have been given; and, finally, that the amount necessary will be that required to neutralise in vitro the poison introduced in an ordinary bite. For the cobra the amount of serum required to save the patient when injected before full absorption of the venom is something over 15 c.c.

**Tetanus.**—In a recent number statistics were quoted showing that some saving of life does occur in acute cases by the use of serum. The following are recent cases; E. Trevithic reports a fatal case in a girl of eight.[11] The length of incubation was unknown, the progress was rapid, and death occurred on the fourth day in spite of the serum. C. Stonham[12] treated a youth in whom symptoms appeared on the sixth day, ending in death in 36 hours. Three doses of serum were given. S. Copley[13] records three cases, in two of which the incubation was from six to ten days, and in the other it was unknown. All these recovered, and the writer insists on the importance of employing large doses of antitoxin. He gives 30 c.c. at once, and repeats them every six hours, and in a severe case he would advise 50 c.c., to be repeated in four hours. Andrew Clark[14] gave serum to a patient whose incubation period was six days, but death followed in 74 hours. Five doses of 10 c.c. each were administered. Galletly[15] refers to a case of his where symptoms came on 19 days after the injury, but increased rapidly. The patient was eight years old, and 20 c.c. were given at first, with others finally amounting to 240 c.c. Complete recovery followed. C. Knox Shaw[16] operated on a young man for fissure of the anus and piles, but five days afterwards tetanus appeared. Serum (20 c.c.) was given, but the patient died in 43 hours. The pus in the ulcer was examined in vain for bacilli, and no other source of infection could be found. The instruments were boiled before use, and other cases operated on by the same surgeon before this one were not infected.

Chauffard and Quenu[17] injected the serum into the brain substance after trephining. The patient had some symptoms as early as four days after an injury, but a complete recovery was obtained. Roux and Borrel had devised this method so as to get the serum to a spot where it can preserve the vital parts of the medulla and cord before they are affected. The operation must be done before the medulla is attacked. Rambaud[18] quotes Bacaloglu's case, which ended fatally after an intracerebral injection, where length of incubation was unknown; a case of Du Hamel's, where incubation lasted 12 days and recovery followed similar treatment; also one by Garnier, incubation unknown, recovery; one by Robert, incubation 13 days, fatal; one by Ombrédaune, incubation nine days, recovery; one by Heckel, incubation unknown, fatal; one by Delmas, incubation seven days, fatal; and Hue's case, incubation six days, fatal. In three of these death occurred in 15 hours, that is before the serum could have any effect. Two fatal cases were also reported by Lucas Championnière under the cerebral method, and one by Martin where lumbar puncture and injection were employed in vain. In N. Robinson's case, 10 days after a laparotomy, the symptoms were removed but the patient sank from her other diseases, while in another American case of Johnson's death took place before the serum could act. C. A. Church, however,[19] gives in detail a successful case where the incubation was 11 days. In this and other instances an injection was made on both sides of the brain to a depth of about two inches, and others were given in various parts of the body. Altogether some 15 cases have been thus treated, of which six recovered, or seven if we include that of Robinson. Brain extract has been shown to have antitoxic powers, and Schram,[20] having prepared some from the brain of a young rabbit, injected it with minute antiseptic precautions in a case of tetanus with successful results. The length of incubation is not, however, given, but symptoms had existed 12 days before this treatment was adopted.

**Rabies.**—F. Cabot[21] has carried out elaborate experiments to discover the protective power of cauterisation after infection from rabies. In guinea pigs 91 per cent. were saved if the wound was cauterised with strong nitric acid within 24 hours, with thorough opening and swabbing out of the wound. The acid was more effective than the actual cautery or nitrate of silver. About 12 per cent. of the animals proved to be immune to rabies. He recommends thorough cauterisation with acid, and opening up the wound in the case of human beings after a bite, whether they receive the Pasteur treatment later on or not.

[1] New York Med. Jour., Feb. 4. [2] Amer. Jour. Med. Sci., Jan. [3] Med. News, Dec. 17. [4] Brit. Med. Jour., Nov. 26. [5] Brit. Med. Jour., Dec. 31. [6] Brit. Med. Jour., Dec. 24. [7] Brit. Med. Jour., April 1. [8] Jour. of Tropical Med., Feb. [9] Intercolonial Med. Jour., Dec. 20. [10] Brit. Med. Jour., April 1. [11] Brit. Med. Jour., Oct. 24. [12] Lancet, Nov. 19. [13] Brit. Med. Jour., Feb. 11. [14] Brit. Med. Jour., Jan. 7. [15] Brit. Med. Jour., Feb. 18. [16] Monthly Homœop. Rev., Mar. 1. [17] Glasgow Med. Jour., Dec. [18] New York Med. Jour., Dec. 17. [19] Ibidem. [20] Med. Rec., Feb. 25. [21] Med. Rec., Jan. 21.

